# Rhabdomyosarcoma-Induced Uterine Inversion

**DOI:** 10.1155/2022/1361803

**Published:** 2022-07-23

**Authors:** Kin Li, Gavin Davis, Colleen Wittenberg, Alireza Abidi

**Affiliations:** ^1^Western University of Health Sciences, College of Osteopathic Medicine of Pacific, Pomona, CA 91766, USA; ^2^Department of OB/GYN, Kaiser Permanente Riverside Medical Center, Riverside, CA 92505, USA

## Abstract

Nonpuerperal uterine inversion is a rare clinical condition that involves prolapse of the uterine fundus into the uterine cavity and vaginal vault and possibly passed the introitus. The majority of these cases commonly involve benign tumors such as leiomyoma. However, another common cause of nonpuerperal uterine inversion is due to malignancies such as sarcomas. Rhabdomyosarcoma is a rare and aggressive malignancy of soft tissue cells that are common in children and rare in adults. One subtype called embryonal rhabdomyosarcoma is exceptionally rare. Therefore, report of embryonal rhabdomyosarcoma-induced uterine inversion is an exceedingly scarce and rarely documented clinical condition. In this case report, we present a rare case of a nulliparous 27-year-old female who presented with embryonal rhabdomyosarcoma-induced uterine inversion.

## 1. Background

Uterine inversion is a rare clinical condition that involves the prolapse of the uterine fundus through the cervix. There are two types of uterine inversions classified as puerperal (obstetrics related) or nonpuerperal (gynecologically related) [1]. Puerperal uterine inversions (PUI) are obstetrical emergencies often associated with high mortality and morbidity from postpartum hemorrhage and hypovolemic shock following delivery or late miscarriages [2]. It has been reported in both acute and chronic cases with an incidence rate of approximately 1 in 3448 deliveries in the US [1, 2].

In comparison, nonpuerperal uterine inversions (NPUI) are more chronic in nature and the true incidence rate is unknown due to its rare nature and lack of systematic reviews and literature aside from case reports [1]. However, unlike PUIs, NPUI are often associated with uterine tumors both benign and malignant. While the most common cause of NPUIs is often a leiomyoma representing 57.5% to 80% of cases, another common cause includes sarcoma representing approximately 13.5% to 20% of cases [1, 3]. Of the different types of sarcomas, embryonal rhabdomyosarcoma represents one of the rarest forms to cause uterine inversions, as there are only a few cases published in literature [4, 5].

Rhabdomyosarcoma (RMS) is generally an aggressively malignant soft tissue tumor that develops from mesenchymal cells. There are four subtypes of rhabdomyosarcoma that include embryonal, alveolar, spindle cell/sclerosing, and pleomorphic [4]. Of these four subtypes, embryonal rhabdomyosarcoma (ERMS) has been reported as one of the rarest forms [5]. RMS is commonly diagnosed in children accounting for approximately 3.4% to 4.2% of all malignancies in children under 10 years old. The most common locations for RMS include the head, neck, limbs, and urinary tract [6, 7]. As age increases, the incidence and rate of RMS becomes rarer as soft tissue sarcomas are only 1% of all adult cancers, which RMS accounts for 3.3-5.0% of all soft tissue sarcomas [6, 7]. The most common location for RMS in adults are generally located in deep soft tissue of the limbs. Compared to other gynecologic malignancies and locations, uterine RMS is an exceptionally rare occurrence with only 3% of RMS occurring in the female genital tract and only a few cases have been reported as ERMS [5, 6]. In this case report, we present an exceptionally rare case of a nulliparous 27-year-old female who presented with ERMS induced uterine inversion.

## 2. Case Presentation

**Figure 1:**
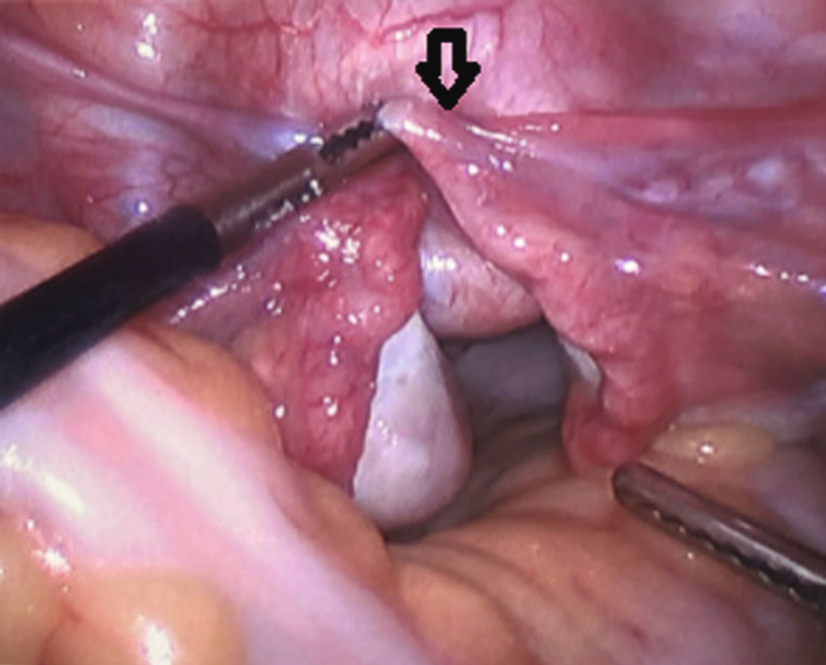
Intraoperative picture of laparoscopic view of inverted fundus prolapsing into the cervix with fallopian tubes and round ligament traction (arrow).

A 27-year-old nulliparous female initially presented with vaginal bleeding, urinary retention, abdominal pain, and a large, necrotic appearing vaginal mass. Initial biopsies returned back as inflammatory exudates with necrotic tissue. However, due to the patients worsening vaginal bleeding and foul vaginal discharge, she was examined under anesthesia for further biopsies and possible uterine inversion repair. On examination, she had a large, friable, necrotic appearing mass in the upper vagina that appeared to be protruding from the cervix. Larger biopsies were performed, and it was determined that a diagnostic laparoscopy was needed. During diagnostic laparoscopy, the fundus was found to be inverted into the cervix pulling with it healthy appearing ovaries and fallopian tubes (Figure 1). After an unsuccessful laparoscopic attempt to revert the uterus to its normal position, it was determined that a laparotomy and vertical hysterotomy were needed to evert the uterus back to its normal position. Any further necrotic appearing tissue was also excised at this point before closure of the hysterotomy.

Histopathology results from the larger biopsies returned back as embryonal rhabdomyosarcoma with positive immunostaining for myogenin and myogenic differentiation 1 (Myo-D1). Due to the aggressive nature of ERMS, she proceeded with a total hysterectomy with bilateral salpingo-oophorectomy a month after diagnosis. Final pathology showed no residual disease. Per tumor board recommendations, patient initiated VAC chemotherapy 1 month after surgery and is currently without any evidence of recurrence.

## 3. Discussion

NPUI are rare clinical occurrences accounting for approximately 1 in 6 or 16.66% of all uterine inversions [3]. However, based on our current literature search, there has only been less than 10 published cases of NPUI caused by ERMS (Table 1) [4, 5, 8]. Current literature has just over 300 cases of NPUI with the majority caused by leiomyomas [1]. While the etiology of PUI has been reported on extensively in previous studies, the etiology of NPUI remains unclear and possibly multifactorial [2]. Previous studies have suggested etiologies such as thin uterine walls from pressure atrophy, traction of large tumor growths located on the fundus, and the dilation of the cervix as possible causes for NPUI [1, 3–6, 9]. In addition, some studies have also reported risk factors including nulliparity, menopause, and uterine contractions that facilitate prolapse of the uterine fundus. Of these risk factors, our patient was nulliparous and had increasingly painful abdominal cramping, which may possibly be related to increased uterine contractions.

Similar to our patient, most women who present with NPUI are often associated with foul-smelling vaginal discharge, severe vaginal bleeding, abdominal discomfort/cramping, and pain [3–5]. In addition, some women also develop urinary retention and hypovolemic shock as some literature has described severe bleeding in women and an initial mean Hgb of 7 g/dL [1, 9]. During the physical examination, it is also important to stage uterine inversions to understand the severity of the condition. Current stages include stage 1 (incomplete inversion with fundus remaining in uterine cavity), stage 2 (complete inversion with uterine fundus through the ring of the cervix), stage 3 (total inversion where fundus protrudes through the vulva), and stage 4 (complete uterine inversion with vaginal involvement through the vulva) [1, 3].

Preoperative diagnosis of NPUI remains difficult, and physicians need to have a high index of suspicion for this clinical condition when patients present with the aforementioned signs and symptoms. The gold standard diagnostic imaging modalities used are ultrasonography and MRI [1, 3, 4, 9]. Previous studies suggested ultrasonographic findings of a depressed longitudinal groove extending from the uterus to the center of an incompletely inverted uterus that creates a “Y”-shaped uterus or a “U”-shaped uterus in the longitudinal plane of completely inverted uterus [1, 3, 9]. In T2-weighted MRI scans, most commonly, a U-shaped uterine cavity with thickened and inverted uterine fundus on sagittal imaging or a “bulls-eye” on axial imaging are suggestive of uterine inversions [1, 3, 9].

Biopsies, frozen sections, and immunohistochemistry are important workups for definitive diagnosis of RMS given the difficult nature of this condition [1, 4]. However, physicians should be aware that biopsies from the superficial tumor may not be able to capture the extent of the tumor due to the extensive necrosis and inflammation usually present. In our case and cases in previous literature, there have been reports of negative results when biopsies were performed on the lower portions of the mass, which can cause misdiagnosis [4, 5, 8]. Instead, it is important to consider larger excisional biopsies to capture the tumor, which may need to be performed under general anesthesia. Commonly used muscle antigen markers for diagnosis of RMS include myogenin, MyoD1, desmin, and sarcomeric actin [6]. In our case, our patient initially had two negative biopsies but a third was positive for MyoD1 and myogenin, which helps confirm skeletal muscle differentiation. Similar to previous literature, in our case, the initial biopsies may be related to inadequate sampling of the tumor, whereas the third was large enough to capture the malignancy.

Proper workup and TNM staging are crucial before definitive surgery, as findings will help with early diagnosis and guide surgical approaches [4]. Currently, a variety of vaginal, abdominal, and combination vaginal and abdominal approaches including Huntingotn, Haultain, Spinelli, and Kustner's operations are reportedly used to treat NPUI with the most common being laparotomies (48.8%) and combination (27.1%) right behind that [3, 5]. However, physicians need to be cautious about cutting vaginal masses during biopsies as previous literature has described cases of inadvertent perforation and dissection of the fundus [1, 4]. Selection of surgical approaches will depend not only on surgeon preference but also the patient's future fertility desires, unique clinical presentations, tumor malignancy, and difficulties encountered during surgery. In cases of malignancy, total hysterectomy is often recommended as returning the inverted uterus to its normal anatomical position may increase the risk of tumor seeding. However, benign tumors such as leiomyomas are excised followed by uterine repositioning and repair when patients wish to retain future fertility [1, 4]. Following surgical correction of NPUI, patients have been reported to have successful pregnancies [1].

The 5-year survival rate for ERMS patients is low at 27%, overall [7]. Several studies have reported that uterine RMS has been associated with poorer prognosis with 47% mortality compared to 8% when it occurs in other sites [4, 6, 11]. In addition, it also seems that ERMS is more aggressive and is associated with an even poorer prognosis as several of the previous literature has reported recurrence of ERMS and mortality within months of recurrence [7]. According to the Intergroup RMS Study Group (IRSG), who studied treatments for RMS in children and adolescents, it recommends a combination of surgery, radiotherapy, and chemotherapy [4]. The gold standard chemotherapy regimen is vincristine, actinomycin-D, and cyclophosphamide (VAC) chemotherapy with or without radiotherapy for 1-10 cycles [4–6, 11]. Due to the rare occurrence in adults, there is no standard treatment regimen established for adult patients. However, studies have shown that similar outcomes were achieved in adult populations following pediatric recommendations for treatment of RMS [4]. In our case, our patient is currently on Vincristine 2 mg, Dactinomycin 2500 mcg, Cytoxan 1200 mg for 10 cycles. She has tolerated the chemotherapy well and without ERMS reoccurrence thus far.

## 4. Conclusion

Nonpuerperal uterine inversions are rare clinical diagnosis that can be caused by malignancies. Physicians should have a high index of suspicion as malignant tumors are the second most common cause for nonpuerperal uterine inversions. Proper workup and TNM staging are important for early diagnosis and prompt treatment. In cases of embryonal rhabdomyosarcoma-induced uterine inversions, the mainstay treatments include a multimodality approach with surgery, VAC chemotherapy, and pelvic radiation therapy.

## Figures and Tables

**Table 1:** Summary of literature with ERMS induced uterine inversions [5, 8, 10].

Authors (year)	Age	Presentation	Findings	Diagnostic imaging	Histopathology	TNM staging	Procedure	Chemotherapy	Outcome
Giacolone et al. (2004)	14 yo female	3 months of postcoidal vaginal bleeding, intermittent pelvic pain, abnormal vaginal discharge, and dyspareunia.	Necrotic mass in the upper vagina and appears to be from the uterine cervix. Hgb 8.5 g/dL	Abdominal U/S-well-define mass of intermediate echogenicity 9 cm MRI of pelvis-polypoidal mass 9 cm in length and pedunculated from uterine fundus projecting into the upper vagina.	Initial biopsy revealed a uterine fibroma with high necrotic component Second biopsy revealed the same findings of uterine fibroma and high necrotic component Third biopsy reveal large focus of tumor necrosis. A mixture of fibroblast cells with clusters of mature cells with distinct cross-striations and mucoid matrix. Positive immunohistochemical staining showed ERMS.	T1, N0, M0	Laparoscopic mass excision and uterine repair Tumor arterial embolisation (femorally)	Unknown regimen	Disease free 2 months later with normal uterus and on chemotherapy
Ojwang et al. (2006)	Unknown	Unknown	10.5 × 9 cm mass	Unknown	ERMS	Unknown	Unknown	Unknown	Unknown
Da Silva (2008)	15 yo nulliparous	3-month history of AUB with 6 days of necrotic tissue discharge	Protruding necrotic mass in the vaginal introitus Skin pallor Hgb 8.3 g/dL Hct 25% WBC 6500/mm^3^.	U/S-volumnious pelvic mass in cervix with heterogenous texture and hypoechoic areas suggestive on necrosis. CT pelvis-voluminous heterogenous mass with solid and cystic areas 11 cm × 9.7 cm	ERMS with myogenin+, desmin+, sarcomeric actin+ IHC atypical cells with prominent nucleoli, eosinophilic and granular cytoplasms consistent with rhabdomyoblasts in myxoid matrix. Superficial myometrial invasion and proliferation of fusiform cells in short interlaced bundles interspersed with atypica cells.	T1b, N0, M0	Total hysterectomy following Haultain method	Vincristine (1.5 mg), doxorubicin (50 mg), cyclophosphamide (750 mg) and actinomycin D (1 mg) Alternating ifosfamide (1 mg), cisplatin (70 mg), and etoposide (150 mg) 3 weekly intervals with pelvic radiotherapy 5000 cGy over 25 days	Died 9 months after surgery due to recurrence and dissemination of disease
Ambreen et al. (2019)	22 yo nulliparous	Lower abdominal pain, AUB, and heavy bleeding for 4 months	Hgb 6 g/dL Skin pallor Abdominal tenderness Protruding fragile necrotic mass approximately 4 × 5 cm Loss of uterine contour and fundal inversion	U/S-4 × 4 cm elongated mass extending down the vagina	ERMS with proliferation of fusiform cells arranged in nests and sheets. Oval cells with scanty cytoplasm, pleomorphic vesicular nuclei and prominent nucleioli +myogenin	Unknown	Polypectomy Total hysterectomy following Haultain method	VAC therapy	18 cm recurrence after the 3rd cycle. Liver metastasis and death 14 months later.
Suneja et al. (2020)	14 yo female	Vaginal bleeding and polypoid mass in vagina	Soft mass filled the vagina Cervix was not felt After 1 week, the mass became visible at the introitus and was growing aggressively to size of 12-14-week gravid uterus Day 3 of the second cycle of chemotherapy, she developed prolapse of the entire tumor mass and inverted uterus	U/S and MRI-9 × 5.8 × 8 cm septate collection in the vagina with inversion of uterus	Initial biopsy revealed mucous, bacterial colonies, anuclear squamous cells, and no tumor cells. Second biopsy also inconclusive with fibrin clots, anucleate squamous cells, and no tumor cells. Third biopsy revealed malignant mesenchymal tumor possibly ERMS.	T1, N0, M0	Total hysterectomy following Haultain method	Vincristine 1.5 mg, adriamycin 50 mg, and cyclophosphamide 750 mg (VAC) for 6 cycles	Disease free 5 years later
Peng et al. (2021)	19 yo nulliparous Chinese female	Unconscious and convulsing on arrival one day before had headaches, nausea, vomiting, seizures, and expressive aphasia 6-month history of AUB with oral contraceptive use and no exposure to drugs or vaccines with thrombosis risk	Mild vaginal bleeding 9 days into hospital stay Severe vaginal bleeding and vaginal pass on the 11th day of hospital stay. Skin and mucosal pallor. Gynecological examination revealed approximately 17 × 15 × 10 cm mass with local congestion, infection, and necrosis. Mass had a large pedicle, and the cervix was palpable or visible. Hgb drop from 10.8 to 6.3 g/dL over 3 hrs.	CT head-venous sinus thrombosis	ERMS with CD10+, CD68+, Desmin+, Ki-67+, MyoD1+, Myogenin+, and vimentin+	Unknown	Emergent cerebrovascular angiography (femorally) Femoral vein intubation with urokinase for 7 days Vaginal mass excision and inadvertent uterine fundus excision on the 11th day Laparoscopic total hysterectomy and resection of fallopian tubes and cervical tissue	Delayed VAC regimen after 43 days since surgery	Recurrence of 5 cm mass in the pelvis Death in 6 months after recurrence of mass and rapid growth (16 cm) despite the 4th cycle of chemotherapy
Current case	27 yo nulliparous female	Presented with 4-month history of AUB and 2 weeks of worsening bleeding. Right lower abdominal pain (sharp) Worsening 3-month history of cramping Foul smelling vaginal discharge	Initial visit to ER, negative HCG, elevated WBC at 15,600, positive urine nitrites and leukocyte esterase with increased neutrophils to 12,540. External hospital and our hospital saw a necrotic appearing mass in the upper vagina. Hgb dropped to 7.0 g/dL at both facilities requiring pRBC during initial stay at external facility and postoperatively at our facility	TVUS showed prominent 1.8 cm endometrium CT of pelvis showed 8 × 8 cm mass in the uterus Postoperative CT of body revealed no metastatic disease	Initial and secondary biopsies revealed necrotic tissue with inflammation Third larger biopsies revealed ERMS with myogenin+, MyoD1 +	T1, N0, M0	Initially, mass excision with conversion to laparotomy for uterine repair After biopsies, laparoscopic total hysterectomy and bilateral salphingo-oophorectomy	VAC therapy	Alive and currently on chemotherapy without recurrence

## Data Availability

No underlying data was used.

## Abbreviations

PUI: Puerperal uterine inversion
NPUI: Nonpuerperal uterine inversion
RMS: Rhabdomyosarcoma
ERMS: Embryonal rhabdomyosarcoma
VAC: Vincristine, adriamycin, cyclophosphamide
PRBC: Packed red blood cells
ISRG: Intergroup RMS Study Group
TNM: Tumor, nodes, metastasis.

## Additional Points

*Synopsis*. A case presentation of a rhabdomyosarcoma-induced nonpuerperal uterine inversion, which is a rather rare cause of uterine inversion.
